# The Concluding Editorial Regarding the Special Issue “Latest Advances in Prosthodontics: Improving Patient-Centered Outcomes”

**DOI:** 10.3390/jcm11195700

**Published:** 2022-09-27

**Authors:** Javier Montero

**Affiliations:** Department of Surgery, Faculty of Medicine, University of Salamanca, 37007 Salamanca, Spain; javimont@usal.es; Tel.: +34-923294500 (ext. 1996)

According to the current holistic concept of health, all patients require a thorough assessment of their physical, psychological, and emotional well-being, not merely a confirmation or classification of disease. Thus, in the context of oral health, the measurement of the impact of oral conditions on patients’ quality of life should be part of the routine evaluation of their oral health needs and dental treatments. The importance of measuring several patient-centered outcomes has been increasingly reported in the literature as a complementary tool for assessing dental interventions.

Since caries and periodontal disease are the most common oral illnesses that, often preceded by a variety of surgical, periodontal, and restorative treatments, can lead to tooth loss, prosthetic needs are commonly associated with certain factors that affect daily living.

The outcomes of the existing distinct prosthetic treatments are variable and cannot be reliably assessed using clinical measures alone. For example, improvement in masticatory function after replacing missing teeth with either fixed or removable dentures may be simultaneously assessed using subjective and objective methods to effectively quantify changes in mastication with both treatment alternatives. In this context, previous experiences have demonstrated that fixed prostheses are better options than removable partial dentures in terms of mastication, after both objective and subjective assessments [1,2]. Those differences were also observed regarding patient satisfaction and their oral-health-related quality of life. However, the worst situation was experienced by those patients treated with complete dentures, because the masticatory function essentially depends on the number of occlusal units (major predictor of mastication). That is the rationale behind the focus on treatment outcomes for fully edentulous patients.

In this Special Issue, it was shown that new complete dentures resulted in significant improvements in chewing ability, patient satisfaction, and oral-health-related quality of life in comparison with baseline conditions, but the standard of care for mandibular edentulism is the implant-retained overdentures because they produce further and faster significant improvements in these afore-mentioned parameters [1]. Moreover, those patient-centered outcomes may even be greater if fixed hybrid complete dentures are inserted instead of removable ones [2]. Nevertheless, the loading protocol seemed to influence the positive self-reported outcomes rather than the objective practical evaluations [1,2].

In this Special Issue, besides overdentures and fixed hybrid dentures, Yoo et al. demonstrated that implant-crown-retained removable partial dentures (IC-RPDs) could also be considered a viable treatment option for those edentulous patients who need few fixed abutments [3]. The results of this very well-performed retrospective study showed that patients treated with IC-RPDs felt more satisfied with their masticatory ability but less satisfied with their aesthetics compared with those treated with implant-retained overdentures on magnet attachments [3].

On the other hand, one of the longest cohort follow-up studies (6 years) regarding the biologically oriented preparation (BOPT) technique was published in this Special Issue [4]. In this paper, both the clinical and patient-centered outcomes of treatment with single zirconia full-coverage crowns on teeth prepared with BOPT were reported, demonstrating that this protocol is predictable in terms of the stability and thickness of the gingival margin and that patients were highly satisfied with the aesthetic outcomes. Given the fact that dental aesthetics is one of the major domains in patient well-being constructs that may even contribute to physical attractiveness, restorative dentistry in the context of aesthetics is hardly challenged. For such reasons, the orthodontic extrusion of compromised or non-restorable teeth has been demonstrated to be a valuable and non-invasive treatment option for maintaining the optimal peri-implant soft and hard tissue architecture to enhance aesthetic outcomes [5]. In this well-developed clinically oriented paper published in this Special Issue, Huang et al. [5] summarized the available literature but acknowledged that more long-term controlled studies are needed for the establishment of a well evidence-based protocol that would recommend appropriate measures on how patients’ treatment affects their quality of life.

The current concept of evidence-based dentistry supplements the best clinical evidence with the experiences and preferences of patients to effectively address their oral health needs.
